# Treating sleep problems in young people at ultra-high-risk of psychosis: study protocol for a single-blind parallel group randomised controlled feasibility trial (SleepWell)

**DOI:** 10.1136/bmjopen-2020-045235

**Published:** 2020-11-10

**Authors:** Felicity Waite, Thomas Kabir, Louise Johns, Jill Mollison, Apostolos Tsiachristas, Ariane Petit, Emma Černis, Daniel Maughan, Daniel Freeman

**Affiliations:** 1 Department of Psychiatry, University of Oxford, Oxford, UK; 2 Oxford Health NHS Foundation Trust, Oxford, Oxfordshire, UK; 3 The McPin Foundation, London, UK; 4 Nuffield Department of Primary Care Health Sciences, University of Oxford, Oxford, UK; 5 Health Economics Research Centre, Nuffield Department of Population Health, University of Oxford, Oxford, Oxfordshire, UK

**Keywords:** mental health, schizophrenia & psychotic disorders, child & adolescent psychiatry, adult psychiatry, sleep medicine, clinical trials

## Abstract

**Background:**

Effective interventions, targeting key contributory causal factors, are needed to prevent the emergence of severe mental health problems in young people. Insomnia is a common clinical issue that is problematic in its own right but that also leads to the development and persistence of psychotic experiences. The implication is that treating sleep problems may prevent the onset of psychosis. We collected initial case series data with 12 young people at ultra-high-risk of psychosis. Post-intervention, there were improvements in sleep, depression and psychotic experiences. Now we test the feasibility of a randomised controlled trial, with a clinical aim to treat sleep problems and hence reduce depression, psychotic experiences, and prevent transition to psychosis.

**Methods and analysis:**

A randomised controlled feasibility trial will be conducted. Forty patients aged 14 to 25 years who are at ultra-high-risk of psychosis and have sleep disturbance will be recruited from National Health Service (NHS) mental health services. Participants will be randomised to receive either a novel, targeted, youth-focussed sleep intervention in addition to usual care or usual care alone. Assessor-blinded assessments will be conducted at baseline, 3 months (post-intervention) and 9 months (follow-up). The eight-session psychological intervention will target the key mechanisms which disrupt sleep: circadian rhythm irregularities, low sleep pressure, and hyperarousal. To gain an in-depth understanding of participants’ views on the acceptability of the intervention and study procedures, 16 participants (n=10 intervention, n=6 control) will take part in qualitative interviews. Analyses will focus on feasibility outcomes (recruitment, retention, and treatment uptake rates) and provide initial CI estimates of intervention effects. Thematic analysis of the qualitative interviews will assess the acceptability of the intervention and trial procedures.

**Ethics and dissemination:**

The trial has received ethical approval from the NHS Health Research Authority. Findings will be disseminated through peer-reviewed publications, conference presentations, and lay networks.

**Trial registration number:**

ISRCTN85601537.

**Strengths and limitations of this study**

- *Importance of the study:* The SleepWell trial is the first randomised controlled test of a sleep intervention in young patients at ultra-high-risk of psychosis.
- *Causal-interventionist paradigm:* This is the first randomised controlled trial to target a single causal factor in the ultra-high-risk group: the randomised controlled design has the aim of lessening sleep disturbance in the intervention group, thereby testing the underlying theory that sleep disruption is a contributory causal factor in the occurrence of psychotic experiences (most likely via reductions in negative affect).
- *Data collection methods:* The study uses mixed methods (interviews, questionnaires and wearable technology) to address the research questions.
- *Follow-up period:* The follow-up period is 9 months; however, the risk of onset of psychosis may be elevated for longer, meaning that we cannot capture all information about transition rates.
- *Control condition:* There is no active control, so it is not possible to determine which elements of the intervention may produce change.

- *Importance of the study:* The SleepWell trial is the first randomised controlled test of a sleep intervention in young patients at ultra-high-risk of psychosis.
- *Causal-interventionist paradigm:* This is the first randomised controlled trial to target a single causal factor in the ultra-high-risk group: the randomised controlled design has the aim of lessening sleep disturbance in the intervention group, thereby testing the underlying theory that sleep disruption is a contributory causal factor in the occurrence of psychotic experiences (most likely via reductions in negative affect).
- *Data collection methods:* The study uses mixed methods (interviews, questionnaires and wearable technology) to address the research questions.
- *Follow-up period:* The follow-up period is 9 months; however, the risk of onset of psychosis may be elevated for longer, meaning that we cannot capture all information about transition rates.
- *Control condition:* There is no active control, so it is not possible to determine which elements of the intervention may produce change.

## Background

### Rationale

The peak age of onset for mental health problems, including psychosis, is between the ages of 14 and 25 years. Three quarters of all lifetime instances of mental health disorders have started by the age of 24.^1^ There is increasing recognition of the need to develop preventative approaches.^2^ We focus on psychosis, with an emphasis on developing interventions that result in both immediate and longer-term improvements by individually targeting developmentally important, transdiagnostic causal factors, that are problematic in their own right and that young patients want treated. Sleep disturbance is one such issue.

In young people, sleep disruption is associated with poor physical and mental health and worse social outcomes, including greater suicide risk, obesity, and lower educational attainment.^3 4^ Yet, sleep problems have been historically understood as either a symptom of psychiatric disorder or a non-specific epiphenomenon (ie, a secondary problem). Recent research has turned this traditional view on its head and instead shown that sleep disruption is one causal factor in the occurrence of psychotic experiences.^5 6^

When sleep is deliberately reduced, in experimental manipulation studies, it results in an increase in psychotic experiences.^7^ Increases in psychotic experiences due to worsening of sleep are mediated by increases in negative affect and related processes (such as worry and negative self-views).^7^ Conversely, successfully treating sleep problems leads to an improvement in psychotic experiences. A large randomised controlled trial (RCT) with 3755 university students found that psychological treatment was highly effective for treating insomnia and led to significant improvements in non-clinical hallucinations and paranoia.^8^ Sleep treatment in this trial also reduced the likelihood that students would reach the threshold for being at ultra-high-risk of psychosis.

In young people at ultra-high-risk of psychosis, sleep problems are widespread, with estimates of prevalence exceeding 75%.^9^ Insomnia and circadian rhythm disruption are associated with the severity of psychotic experiences and poor outcomes.^10–13^ Findings from a large European prospective multicentre study indicate that sleep problems are highly predictive of transition to psychosis.^14^ In addition, sleep disturbance is also a contributory factor across a wide range of other mental health problems, including anxiety, depression, and bipolar disorder.^6 15^ Therefore, treating sleep problems may also prevent other mental health problems, such as depression and anxiety, and promote improvements in functioning and physical health.

The key diagnostic marker for identifying young people at ultra-high-risk of psychosis is the presence of attenuated (subthreshold frequency or intensity) psychotic experiences (APE). Yet APE are a marker of potential risk for a number of different serious mental health problems, including personality and mood disorders.^16^ Even for young people who experience APE but do not go on to meet diagnostic criteria for psychosis (or other serious mental health problems) the long-term outcomes can remain poor.^17 18^ The clinical staging model in psychiatry emphasises that less differentiated early phases of mental health problems may benefit from broad spectrum and simpler treatments.^19–21^ From a network perspective, mental health problems arise from the causal interplay (both direct and reciprocal) between different symptoms.^22^ Therefore, targeting one specific symptom, such as sleep disturbance, will have an impact on other causally connected problems, such as low mood, anxiety, and psychotic experiences.

Cognitive Behavioural Therapy for insomnia (CBTi) is the first-line treatment for adults with insomnia^23 24^ and has been effectively adapted for people with psychosis.^5 25 26^ Not only does CBTi lead to large improvements in insomnia, but there are also significant effects on depression and anxiety,^8 25 27^ and smaller effects on psychotic experiences.^8 25^ It is likely the treatment effects on psychotic experiences are mediated by improvements in depression and anxiety.^8 27^ In light of this previous work, we now want to test the effects of treating sleep problems to reduce psychotic experiences and prevent the onset of psychosis in those at highest risk. We are also interested in whether this treatment focus reduces emotional disorders both in the short and long-term.

Building on the principles of CBTi and our work treating insomnia in adults with psychosis, we developed a brief psychological intervention to address sleep problems in young people at ultra-high-risk of psychosis (SleepWell).^28^ The core treatment techniques include stimulus control, circadian realignment, and regulating daytime activity. In an uncontrolled case series with 12 young people at ultra-high-risk of psychosis, outcome assessments were conducted pre-treatment, post-treatment, and at a 1-month follow-up. Following the intervention, there were improvements in sleep (*d*=6.8), depression (*d*=0.5), and psychotic experiences (paranoia *d*=0.6 and hallucinations *d*=0.3) and the changes were maintained at the 1-month follow-up. Throughout the 10-week study period, no participants transitioned to psychosis. In the opposite direction, at the end of the study three participants no longer met criteria for being at ultra-high-risk of psychosis. The treatment was popular (for example, 89% attendance rate) and patients were keen to participate. In qualitative interviews, participants described achieving meaningful change in both sleep and well-being by developing a ‘repertoire of skills’.^29^ The treatment now requires testing in a randomised trial to determine the effects on psychotic experiences and the potential to prevent the onset of psychosis.

Due to limitations in statistical power, we will investigate the effects of the sleep treatment on the dimension of psychotic experiences rather than relying solely on the dichotomous outcome of transition. In line with the findings from all previous studies, we expect that the effect of improving sleep on psychotic experiences will be through improvements in negative affect (depression and anxiety), which is a mediator we are also interested in as an outcome in itself.

### Objectives

We aim to assess the feasibility of a targeted intervention to improve sleep and prevent psychosis in young people at ultra-high-risk of psychosis.

The primary objective is to assess the feasibility and acceptability of a targeted sleep intervention to prevent psychosis in young people at ultra-high-risk in order to establish the key parameters for a definitive RCT. The secondary research objective is to gather data on clinical outcomes to provide a preliminary indication of the clinical efficacy of the sleep intervention (SleepWell) for young people attending National Health Service (NHS) mental health services with sleep problems who are at ultra-high-risk of psychosis. Table 1 provides a summary of the objectives (and the associated feasibility markers or assessment measures).

**Table 1:** Summary of objectives and assessment measures

	Objectives	Outcome measures
Primary	Assess the feasibility and acceptability of a targeted sleep intervention to prevent psychosis and to establish key parameters for a future randomised controlled trial.	Number of patients identified, recruited, declined and retained.
		Number of referrals made per site, and per service type, per month.
		Service use as measured on the CSRI.
		Completion rate of each assessment measure, including wearable-technology devices. Time taken to complete each assessment.
		Location and attendance at treatment sessions; content covered in treatment sessions; feedback from qualitative interviews; treatment acceptability score (AARP).
		Service use data completeness, time taken to collect service use data.
Secondary	Gather data on clinical outcomes to provide a preliminary indication of the clinical effectiveness of a novel sleep intervention (SleepWell) in addition to usual care versus usual care alone in young people with sleep problems who are at ultra-high-risk of psychosis attending NHS mental health services	Sleep disturbance: ISI; Sleep-50 CRD; Sleep diary; Actigraphy; Fatigue. Attenuated psychotic experiences: CAARMS; SPEQ-H; R-GPTS; ČEFSA. Psychiatric symptoms: DASS-21; C-SSRS; DWQ; BCSS. Activity and social functioning: Time budget; WASA; Oxford Agoraphobic Avoidance Scale; actigraphy. Physical health: BMI; step-count; BESAA; PHQ15; MAP. Quality of life: QPR; ReQoL; EQ5D. Service use: CSRI; and medication. Participant ranking of clinical outcome variables.
Qualitative	To explore the acceptability of study procedures to service users.	Feedback from qualitative interviews.

AARP, Abbreviated Acceptabliity Rating Profile; BCSS, Brief Core Schema Scale; BESAA, Body Esteem Scale for Adults and Adolescents; CAARMS, Comprehensive Assessment of At-Risk-Mental States; CRD, Circadian Rhythm Disruption; CSRI, Client Service Receipt Inventory; C-SSRS, Columbia Suicide Severity Rating Scale; DASS-21, Depression, Anxiety, and Stress Scale; DWQ, Dunn Worry Questionnaire; ČEFSA, Černis Felt Sense of Anomaly Scale; ISI, Insomnia Severity Index; MAP, Maudsley Addiction Profile; NHS, National Health Service; PHQ15, Patient Health Questionnaire; QPR, Process of Recovery Questionniare; R-GPTS, Revised Green et al Paranoid Thoughts Scale; SPEQ-H, Specific Psychotic Experiences Questionnaire - Hallucinations subscale; WASA, Work and Social Adjustment Scale.

Our hypotheses related to clinical outcomes are:

1. Compared with usual care, the SleepWell therapy added to usual care will reduce insomnia and other sleep disruption (post-treatment).
2. Compared with usual care, the SleepWell therapy added to usual care will reduce psychotic experiences (a key marker of psychosis risk) and rates of transition to psychosis (post-treatment).
3. Compared with usual care, the SleepWell therapy added to usual care will reduce psychiatric symptoms (depression, anxiety, worry, suicidal ideation), increase activity and social functioning, improve physical health, and enhance quality of life (post-treatment).
4. Treatment effects will be maintained at follow-up.

## Methods and analysis

### Trial design and flow chart

**Figure 1:**
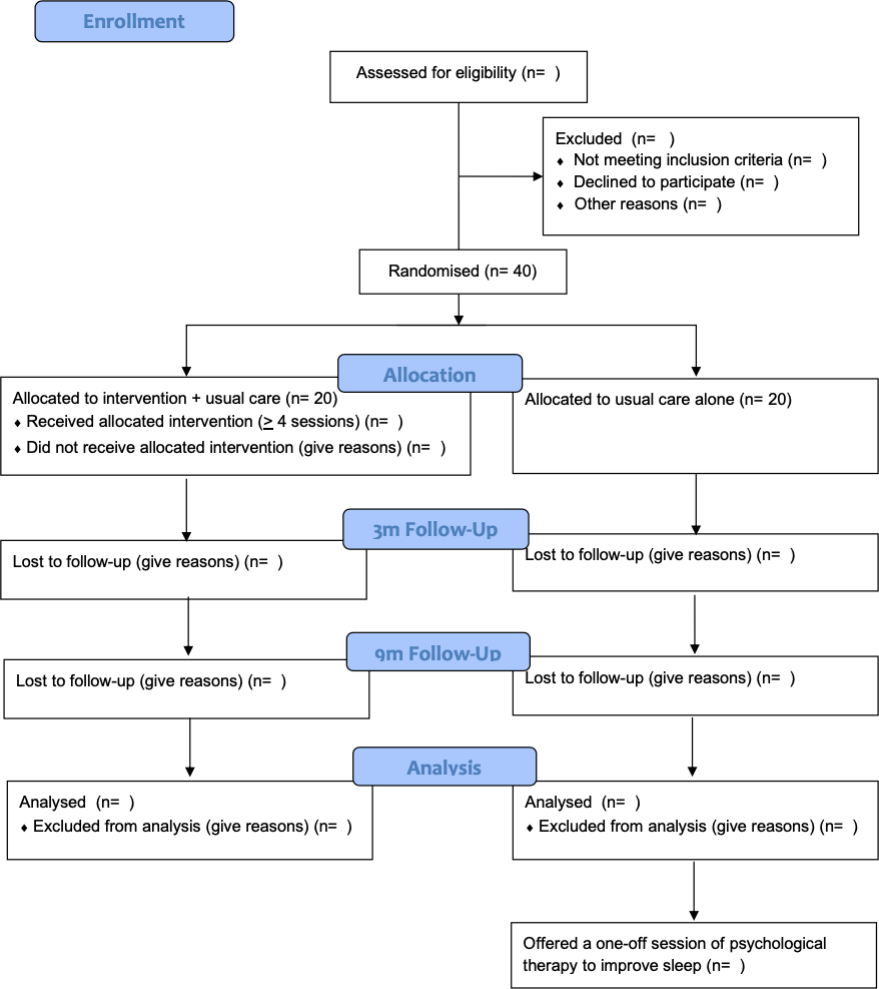
Trial flow diagram.

The design is a prospective, parallel group, single blind, randomised controlled feasibility trial to evaluate a novel sleep intervention (SleepWell) in addition to usual care versus usual care alone in young people with sleep problems who are at ultra-high-risk of psychosis and attending NHS mental health services. Standard care will be measured, but remain as usual in both groups. Assessments will be carried out at 0 (baseline), 3 (post-treatment) and 9 (follow-up) months by a researcher blind to treatment allocation. An embedded qualitative study will explore the acceptability of study procedures to participants. A summary of the trial design can be seen in figure 1. The trial is prospectively registered with the ISRCTN registry: ISRCTN85601537. There is a trial Data Monitoring and Ethics Committee (DMEC) and Lived Experience Advisory Panel (LEAP). The LEAP is facilitated by the McPin Foundation.

### Randomisation, blinding and code-breaking

Participants will be randomised once they have completed the baseline assessment. Participants will be allocated to one of the trial arms using a 1:1 allocation ratio. Randomisation will be carried out by a validated online system, Sortition, designed by the University of Oxford Primary Care Clinical Trials Unit. Allocation will be carried out using a non-deterministic minimisation algorithm to ensure balance across groups with respect to severity of sleep disturbance (Insomnia Severity Index (ISI) score ≤21/≥22) and referring service (early intervention in psychosis team (EIP), child and adolescent mental health services (CAMHS), improving access to talking therapies service (IAPT)).

The research assessor will be blinded to group allocation, but the patients and clinician delivering the intervention will not be (they cannot be blinded to whether a psychological intervention is delivered or received). If an allocation is revealed between assessment sessions, this is logged by the trial coordinator and future assessments conducted, where possible, by another assessor to re-establish the blind.

### Participants

The trial participants will be 40 young people (aged 14 to 25 years) attending NHS mental health services with sleep problems who are at ultra-high-risk of psychosis. The principal method of recruitment will be via seeking referrals from the relevant clinical teams in the participating NHS mental health trusts. The services include specialist early intervention in psychosis services (EIP: ages over 14 years), child and adolescent mental health services (CAMHS: ages 0 to 18 years) and improving access to psychological therapies services (IAPT: ages over 18 years). The study will be advertised within participating NHS trusts and on social media so that young people receiving care in the recruiting NHS trusts can self-initiate referral to the trial. With the approval of the clinical team, all young people interested in taking part will then be approached by the research team, given information about the trial, and the screening conducted. The importance of self-initiated referral was highlighted by our LEAP to ensure accessibility and inclusivity. Informed consent will be obtained from all patients before participation.

#### Inclusion criteria

- Aged 14 to 25 years;
- Patient of mental health services (at the time of referral to the study);
- Meet diagnostic criteria for ultra-high-risk of psychosis on the Comprehensive Assessment of At-Risk-Mental-States (CAARMS);^30^
- Experiencing current sleep problems (defined as a score >15 on the Insomnia Severity Index (ISI)^31^);
- Would like help to improve sleep; and
- Willing and able to give informed consent (or assent with parent/guardian consent for participants aged 14 to 15 years) for participation in the trial.

#### Exclusion criteria

- Diagnosis of a primary severe mental health problem (including psychosis, bipolar disorder, personality disorder);
- Likely primary diagnosis of sleep apnoea (established using the STOP-BANG screen^32^);
- A primary diagnosis of alcohol or substance disorder;
- Organic syndrome;
- Significant learning disability; or
- Current engagement in any other individual psychological therapy.

### Assessments

Basic demographic and clinical data will be collected (eg, age, gender, ethnicity, clinical diagnosis). Clinical outcomes including sleep disturbance, psychotic and affective symptoms, social functioning, quality of life and physical health will be assessed at all three time points (baseline 0 months, post-intervention 3 months and follow-up 9 months).

Insomnia (ISI),^31^ circadian rhythm disruption (SLEEP-50 CRD subscale^33^) and fatigue^34^ will be assessed via self-report. Sleep and activity levels will also be assessed using actigraphy (over 7 days), complemented with sleep diaries and a time-budget assessing meaningful activity.^35^

Attenuated psychotic symptoms and transition to psychosis (Comprehensive Assessment of At-Risk-Mental-States (CAARMS)^30^), hallucinatory experiences (Specific Psychotic Experiences Questionnaire – Hallucinations subscale (SPEQ-H)^36^), paranoia (Revised- Green *et al* Paranoid Thoughts scale (R-GPTS)^37^) and dissociative experiences (Černis Felt Sense of Anomaly Scale (ČEFSA)^38^) will be assessed.

Levels of depression and anxiety (Depression, Anxiety and Stress Scale (DASS-21)^39^), worry (Dunn Worry Questionnaire (DWQ)^40^), suicidal ideation (Columbia Suicide Severity Rating Scale (C-SSRS)^41^) and self-concept (Brief Core Schema Scale (BCSS)^42^) will be measured.

Social functioning will be assessed using the Work and Social Adjustment Scale (WASA)^43^ and Oxford Agoraphobic Avoidance Scale.^44^ The Process of Recovery Questionnaire (QPR),^45^ EQ-5D-5L (http://www.euroqol.org/) and ReQol^46^ will assess quality of life.

Body mass index (BMI), somatic symptoms (Patient Health Questionnaire 15 (PHQ15)^47^), appearance concerns (Body Esteem Scale for Adults and Adolescents (BESAA)^48^ and substance use (Maudsley Addiction Profile (MAP)^49^) will be measured as markers of physical health. Service use, and other relevant health economic data, will be recorded using the Client Service Receipt Inventory (CSRI).^50^

A qualitative interview will be conducted with a subset of 16 participants (n=10 intervention, n=6 control) after their final assessment. The semi-structured interview will explore participants’ experiences of therapy and trial participation.

### The SleepWell intervention

SleepWell is a psychological intervention designed for young people that targets the key mechanisms which regulate sleep: circadian rhythm, sleep pressure, and hyperarousal.^51–53^

1. To target circadian rhythm, the timing of sleep, we use light/dark exposure which is the key zeitgeber or time cue for sleep, to re-align sleep patterns with the environment. We also re-establish circadian rhythms using daily activity points.
2. To target sleep pressure, the need for sleep, we increase daytime activity to increase night-time tiredness. We use the motivational benefits of fitness-trackers.
3. To target hyperarousal, which can disrupt sleep despite circadian entrainment and high sleep pressure, the key strategy is stimulus control which enables patients to relearn the association between bed and sleep. We also use worry reduction strategies, cognitive restructuring techniques, and night-time relaxation.

SleepWell has been specifically designed with consideration of the unique aspects of sleep in youth, such as the biological changes in sleep architecture (eg, delayed sleep-phase) and lifestyle factors (eg, exam pressures, increasing independence). For example, SleepWell includes the use of technology-devices, engaging family/friends to support the young person, and adaptations of stimulus control due to environmental constraints such as shared accommodation at university.

The SleepWell intervention is manualised in a modular format. The five core modules include: (1) psychoeducation, assessment and goal setting (eg, actigraphy data are used to monitor sleep patterns and identify foci for change); (2) establishing the environmental and lifestyle context for sleep; (3) stimulus control and strategies to reduce hyperarousal; (4) circadian entrainment (using light/dark exposure, setting the sleep window, boosting zeitgebers, for example, meal and activity times); and (5) relapse prevention. Additional modules are selected by patients on the basis of individual need, enabling the intervention to be personalised to the individual’s needs (eg, night-time worry). The format and manuals have been developed in collaboration with our LEAP.

The intervention is delivered by a clinical psychologist, on an individual basis in up to eight 1-hour sessions. There is typically one session per week. Additional contact between sessions (eg, text messages, email) is provided to support treatment strategy implementation. A treatment dose is defined as four or more sessions. In line with the International Early Psychosis Association recommendations that interventions are provided flexibly and in low stigmatising environments, sessions are held in the patient’s home or local health service clinic.^54^ At the final session, participants will be asked to complete a self-report questionnaire to assess the acceptability of the intervention (AARP^55^).

SleepWell will be provided in addition to usual care. With patient consent, sessions will be audio-recorded and independently rated for quality, including fidelity and competence.

### Control condition

Participants who are allocated to the control arm will continue to receive their usual care. Treatment as usual for the participants in this trial will typically consist of monitoring meetings with a general practitioner or mental health practitioner and psychiatric medication (as needed). Treatment as usual will vary across individuals, clinical teams and mental health trusts. We will collect detailed data on treatment as usual (which will also inform the preliminary health economic evaluation).

At the end of their participation in the study, participants in the control arm will be offered a one-off session about sleep with a clinical psychologist. This session will briefly identify a plan to improve sleep, which participants may then implement independently. The LEAP emphasised the importance of offering this session.

### Adverse events

A serious adverse event (SAE) is defined as any untoward medical occurrence that results in death, is life threatening, requires or prolongs hospitalisation or results in persistent or significant disability/incapacity. The sorts of SAEs that can typically occur in this participant group include: deaths, suicide attempts, serious violent incidents, and admissions to hospital. Hospitalisation for a pre-existing physical health condition, including elective procedures planned prior to study entry, which has not worsened, does not constitute an SAE.

A trial standard operational procedure has been written for adverse events. We will record the occurrence of any SAEs reported to us and also systematically check all participants’ medical records following completion of the final assessment. We will also record transition to psychosis and formal complaints regarding therapy. Transition to psychosis will be determined through multiple sources including: scoring above psychosis threshold on the CAARMS^30^ at the research assessments; review of medical records; clinical team feedback; prescription of anti-psychotic medication, and the clinical observation of the study therapist. The responsible clinical team, the trial management committee and the DMEC will be informed of any adverse event.

### Analysis

A full statistical analysis plan will be drafted prior to recruitment beginning and finalised before any analysis takes place. There are no interim analyses or formal stopping rules in relation to this study as the primary goal is to establish feasibility parameters for a definitive trial. Data will be reported in line with the CONSORT 2010 Statement, SPIRIT and GRIPP2 guidelines.

Analysis will be descriptive in nature and no hypothesis testing will be carried out. The number (percentage) will be presented for feasibility measures (ie, recruitment and retention, uptake of treatment, data completion) overall and by randomised group. Progression criteria related to key feasibility outcomes such as recruitment, retention, and treatment uptake rates have been identified. These criteria outline the potential progression from feasibility to definitive trial. Each criterion has ‘stop/amend/go’ indicators. For example, the progression criterion for treatment uptake will be: 75% and above indicates the progression criterion has been met, 51% to 75% indicates the need to amend, and below 50% indicates stop.

The patient outcome measures will be described using the mean (SD) or median (IQR) depending on the distribution of the measure and by the number (percentage) for binary outcomes. At baseline, patient measures will be reported overall and by randomised group. At follow-up points, patient outcomes will be presented by randomised group and the difference between groups and 95% CI for the difference will be reported to aid sample size calculations for the definitive trial.

The primary aim of this feasibility trial is to establish the necessary parameters for a definitive trial. This includes recruitment, retention, and treatment uptake rates. A sample size of 20 per randomised group (40, in total across the study) will be sufficient to estimate a recruitment rate of 50% with a 95% CI of 35% to 65% and a retention rate of 80% with a 95% CI of 65% to 90% (PASS V.12). This sample size of 20 per arm will be sufficient to estimate the variability of outcome measures for future sample size calculations, with 12 per arm sufficient for estimation of the variability for the purpose of sample size calculations.^56^

All participants recruited to the feasibility trial will be documented fully with respect to receiving the intervention and participating in follow-up. Although no formal statistical analysis will be undertaken, outcomes will be reported by randomised group for all participants randomised, irrespective of whether they received the intervention or not (intention-to-treat). Adverse events will be reported for all participants randomised.

A microcosting approach will be used to inform the cost per patient of the SleepWell intervention. As part of this feasibility study, we will determine the acceptability and completeness of the necessary data (CSRI, medication use, ReQOL and EQ-5D) to perform a full health economic analysis. Further to this we will investigate the possibility of developing a decision-analytical model that would permit the generation of cost-effectiveness estimates in the long-term (eg, costs and outcomes in patient’s lifetime) based on a large evaluation trial of the SleepWell intervention.

The qualitative interviews will be recorded, transcribed verbatim, and analysed using Thematic Analysis.^57^ This includes the identification of codes, candidate themes and thematic map. A thematic map reflects the meanings of the overall data set and provides a conceptualisation of the themes and relationships between them. Quality guidelines^58 59^ will be followed, including credibility checks and reflexive practice.

### Patient and public involvement

Patient and Public Involvement is being facilitated by The McPin Foundation, a charity that exists to ‘put the lived experience of people affected by mental health problems at the heart of research’ (www.mcpin.org). A grant holder is from The McPin Foundation. The application was developed in collaboration with young people with experience of sleep problems and psychosis. Following the award of the grant, a fully funded LEAP has been formed to advise on all stages of the research programme (for example, participant recruitment, analysis of the qualitative interview data and dissemination of the research findings). The LEAP will meet throughout the course of the trial.

For the trial protocol, the LEAP has advised on: the length of follow-up period, the age range of participants, the choice of outcome measures, recruitment methods, the format of recruitment materials and the content and wording of study materials (including the information sheet, consent/assent form, and therapy manuals). The LEAP has also reviewed and commented on the trial protocol document.

### Ethics and dissemination

The trial has received Health Research Authority (HRA/HCRW) approval (IRAS 281235, The SleepWell trial). The trial received ethical approval from the NHS South Central - Oxford A Research Ethics Committee (20/SC/0281). R&D teams at participating NHS trusts will confirm local capacity and capability to deliver the research. The University of Oxford is the trial sponsor. The results of the trial will be published in a peer-reviewed journal and made open access. An anonymised version of the main outcome data will be available from the trial team on reasonable request after publication of the main results paper. A summary of the results, developed in collaboration with the LEAP, will be provided to all participants.

### Trial status

The trial is due to start patient recruitment in November 2020. Recruitment will be for 14 months until January 2022, with final outcome data collected by September 2022. A trial paper detailing the outcomes should be submitted for publication around December 2022.

## Supplementary Material

Reviewer comments
