# Supplementary material for: Treating sleep problems in young people at ultra-high-risk of psychosis: study protocol for a single-blind parallel group randomised controlled feasibility trial (SleepWell)
Source: BMJ Open. 2020 Nov 10;10(11):e045235. doi: 10.1136/bmjopen-2020-045235 (PMC7656948; doi:10.1136/bmjopen-2020-045235)
Supplement: Reviewer comments [file bmjopen-2020-045235.reviewer_comments.pdf]

PEER REVIEW HISTORY

BMJ Open publishes all reviews undertaken for accepted manuscripts. Reviewers are asked to complete a checklist review form (**http://bmjopen.bmj.com/site/about/resources/checklist.pdf**) and are provided with free text boxes to elaborate on their assessment. These free text comments are reproduced below.

ARTICLE DETAILS

**TITLE (PROVISIONAL)**	Treating sleep problems in young people at ultra-high-risk of psychosis: study protocol for a single-blind parallel group randomised controlled feasibility trial (SleepWell)
**AUTHORS**	Waite, Felicity; Kabir, Thomas; Johns, Louise; Mollison, Jill; Tsiachristas, Apostolos; Petit, Ariane; Černis, Emma; Maughan, Daniel; Freeman, Daniel

This article was not externally reviewed at BMJ Open. Protocols that have been independently assessed prior to submission to BMJ Open are usually fast-tracked to publication on the grounds that further substantial changes will not be possible. This independent assessment will usually be external, independent review for both a substantial grant award from a non-commercial or government funder and ethics approval.
